# Habituation or sensitization of brain response to food cues: Temporal dynamic analysis in an functional magnetic resonance imaging study

**DOI:** 10.3389/fnhum.2023.1076711

**Published:** 2023-02-17

**Authors:** Peyman Ghobadi-Azbari, Rasoul Mahdavifar Khayati, Hamed Ekhtiari

**Affiliations:** ^1^Department of Biomedical Engineering, Shahed University, Tehran, Iran; ^2^Department of Psychiatry, University of Minnesota, Minnesota, MN, United States

**Keywords:** temporal dynamic, fMRI, cue-reactivity, craving, habituation

## Abstract

**Introduction:**

In the modern obesogenic environment, heightened reactivity to food-associated cues plays a major role in overconsumption by evoking appetitive responses. Accordingly, functional magnetic resonance imaging (fMRI) studies have implicated regions of the salience and rewards processing in this dysfunctional food cue-reactivity, but the temporal dynamics of brain activation (sensitization or habituation over time) remain poorly understood.

**Methods:**

Forty-nine obese or overweight adults were scanned in a single fMRI session to examine brain activation during the performance of a food cue-reactivity task. A general linear model (GLM) was used to validate the activation pattern of food cue reactivity in food > neutral contrast. The linear mixed effect models were used to examine the effect of time on the neuronal response during the paradigm of food cue reactivity. Neuro-behavioral relationships were investigated with Pearson’s correlation tests and group factor analysis (GFA).

**Results:**

A linear mixed-effect model revealed a trend for the time-by-condition interactions in the left medial amygdala [t(289) = 2.21, β = 0.1, *P* = 0.028], right lateral amygdala [t(289) = 2.01, β = 0.26, *P* = 0.045], right nucleus accumbens (NAc) [t(289) = 2.81, β = 0.13, *P* = 0.005] and left dorsolateral prefrontal cortex (DLPFC) [t(289) = 2.58, β = 0.14, *P* = 0.01], as well as in the left superior temporal cortex [42 Area: t(289) = 2.53, β = 0.15, *P* = 0.012; TE1.0_TE1.2 Area: t(289) = 3.13, β = 0.27, *P* = 0.002]. Habituation of blood-oxygenation-level-dependent (BOLD) signal during exposure to food vs. neutral stimuli was evident in these regions. We have not found any area in the brain with significant increased response to food-related cues over time (sensitization). Our results elucidate the temporal dynamics of cue-reactivity in overweight and obese individuals with food-induced craving. Both subcortical areas involved in reward processing and cortical areas involved in inhibitory processing are getting habituated over time in response to food vs. neutral cues. There were significant bivariate correlations between self-report behavioral/psychological measures with individual habituation slopes for the regions with dynamic activity, but no robust cross-unit latent factors were identified between the behavioral, demographic, and self-report psychological groups.

**Discussion:**

This work provides novel insights into dynamic neural circuit mechanisms supporting food cue reactivity, thereby suggesting pathways in biomarker development and cue-desensitization interventions.

## Introduction

Overweight and obesity affect 39% of the adult population, are a leading cause of death in the adults and are considered a public health crisis in the USA and other countries, with more than 1.9 billion adults suffering from overweight, of which 650 million are obese (Ritchie and Roser, 2017). In light of the dramatically rising mortality and morbidity with surplus body weight (Hruby and Hu, 2015; Abdelaal et al., 2017), which is related to the overeating of highly palatable foods, it is critical to understand the physiology of overweight and obesity and the neurocognitive architecture underlying relevant behaviors. Food craving is driven by the presence of stimuli that predict the rewarding effect of food (Robinson and Berridge, 1993; Nederkoorn and Jansen, 2002; Pedersen et al., 2022), which is a key feature of eating disorders, but its association with medical and psychological outcomes might depend on the type of eating disorder (Chao et al., 2016; Oliveira and Cordás, 2020).

The incentive-sensitization theory of obesity postulates that repeated pairings of rewards from food intake and cues that predict imminent food intake result in a hyper-responsivity of rewards circuitry to food cues, which can lead to craving and overeating (Berridge et al., 2010; Nijs and Franken, 2012; Polk et al., 2017; Agarwal et al., 2021). Food craving and subsequent weight gain can be triggered by exposure to cues associated with palatable foods (Boswell and Kober, 2016). The cue-potentiated feeding response is the outcome of conditioning, in which neutral stimuli take on incentive value after being repeatedly paired with food ingestion (Han et al., 2018). Such cues include the sight, smell, and taste of food. As an “ecologically valid” approach, food cue exposure has been developed increasingly as a proxy measure for eliciting cue reactivity and craving to examine the mechanistic and predictive value of subjective, behavioral, and biological responses to food cues (Lawrence et al., 2012; Boswell and Kober, 2016; Hermann et al., 2019; Ghobadi-Azbari et al., 2022; Kanoski and Boutelle, 2022).

Functional magnetic resonance imaging (fMRI) is a standard tool to investigate the neural correlates of food cravings during cue exposure. These studies reported that neural cue reactivity as indexed by fMRI signals is prospectively associated with food seeking/consumption behavior measured by weight gain (Demos et al., 2012; Yokum et al., 2014) but also predictive of weight loss intervention outcome (Hermann et al., 2019; Neseliler et al., 2019).

Most fMRI food cue reactivity studies model the response to a repeated stimulus using a single constant-amplitude regressor, an approach that provides an estimate of the average response amplitude along the fMRI session. However, there is a shred of evidence that cue reactivity triggers a motivational response that has a temporal behavior (Fischer et al., 2003; Strauss et al., 2005; Williams et al., 2013; Murphy et al., 2018; Ekhtiari et al., 2021; Jafakesh et al., 2022). In accordance with conventional methodological guidelines for fMRI cue-reactivity paradigms, multiple exposures to cues occur as blocks or events throughout each experiment. This provides a fantastic opportunity to study the temporal dynamics of the response to food cues by include time in the analysis. During repeated cue exposure trials, we anticipate that various regions will exhibit habituating or potentiating responses. Previous fMRI cue reactivity studies have almost exclusively focused on dynamic responses to affective cues (Wright et al., 2001; Luan Phan et al., 2003), including brain areas such as the amygdala, ventral striatum, nucleus accumbens, and prefrontal cortex. In particular, the amygdala, a region commonly associated with rewards valuation and emotional reactivity, rapidly habituates to repeated exposure to emotional stimuli (Breiter et al., 1996; Fischer et al., 2003; Sladky et al., 2012; Plichta et al., 2014). In addition, the nucleus accumbens habituates as the salience of the motivational stimuli decreases with repetition (De Luca, 2014). Several studies have demonstrated apparent habituation effects to repeated presentations of drug cues in the superior temporal gyrus (Ekhtiari et al., 2021; Jafakesh et al., 2022). Of particular interest from the behavioral and clinical perspective, superior temporal gyrus habituation has been shown to be negatively correlated with omission error and reaction time (Jafakesh et al., 2022). Furthermore, it is correlated with drug cravings, the total number of risky behaviors, and the number of abuse days in the last month. However, the habituation/sensitization of a neural response to repeated exposure to food vs. neutral stimuli within overweight and obese people remain unclear. This study intends to examine the temporally dynamic brain activation patterns that underlie food cue-reactivity in overweight and obese people using data from the fMRI implementation of a food cue-reactivity task. The activation slopes may reflect the sensitization (positive slope) or habituation (negative slope) of brain regions as they engage in the processing of food cues. In addition, the clinical and behavioral correlates of activation slopes over time in regions with time-varying activity will be investigated, providing an initial estimate of the potential clinical utility of activation slopes. To our knowledge, this is the first food cue-reactivity study using a sliding window over the BOLD response to assess the temporal dynamics of brain activation.

## Materials and methods

### Participants

The study database was the same as described in our previous work (Ghobadi-Azbari et al., 2022). After giving written informed consent, 54 obese or overweight volunteers participated in the fMRI experiment based on previous calculations of statistical power. A total of five participants were excluded from the study—two for excessive head motion during scanning, two for unanticipated claustrophobia, and one for intracranial lesion. Thus, data from 49 participants were analyzed (33 females; mean age 35.55 years, range 21–59 years). Table 1 provides information on demographic and behavioral characteristics. Participants filled out a screening questionnaire to assess eligibility for the study. Interested individuals were eligible if they reported an unhealthy BMI (26–35 kg/m^2^), no current or past history of psychiatric or neurological disorders, no current or past history of eating or substance use disorders, no current psychopharmacological medication, no current pregnancy, and meeting MR-specific inclusion criteria (lack of claustrophobia, no metallic implants, etc.). All participants had normal or corrected-to-normal vision. As we aimed to study food cue reactivity in a sample of overweight/obese adults who have frequent food cravings (≥3 per day during last month), we also excluded people who (1) reported having severe food allergies, (2) reported having special diets, (3) reported gastrointestinal disorders, metabolic, or endocrine disease, or (4) reported lower sensitivity to food cue responsiveness (i.e., people with mean craving scores <80). The study was approved by the ethics committee of the Iran University of Medical Sciences (IR.IUMS.REC.1396.0459).

**TABLE 1:** Sample characteristics (*n* = 49).

Variable	Mean	SD
Age	35.55	10.00
Height (m)	1.67	0.10
Weight (kg)	82.57	14.51
BMI (kg m^–2^)	29.60	3.63
Education (year)	16.88	2.33
CES	10.94	5.81
**DASS**	20.77	12.77
Depression (0–21)	6.13	4.97
Anxiety (0–21)	5.02	4.15
Stress (0–21)	9.63	5.06
**TFEQ**		
Hunger (0–30)	14.44	5.43
Cognitive restraint (0–12)	5.69	2.78
Emotional eating (0–6)	3.44	1.61
**EDDQ**		
Body image (0–24)	14.69	5.39
Overeating (0–8)	4.44	2.08
Compensatory behaviors (0–56)	3.19	4.08
**FCQ-trait**	89.54	39.70
Lack of control under environmental cues	28.50	14.85
Thoughts or preoccupation with food	12.25	8.46
Hedonic hunger	30.88	13.22
Emotions before or during food craving	10.92	5.61
Guilt from craving	7.00	4.32
**FCQ-state**	31.91	16.04
Intense desire to eat	7.52	3.92
Anticipation of positive reinforcement that may result from eating	7.77	3.86
Anticipation of relief from negative states and feelings as a result of eating	6.07	4.07
Obsessive preoccupation with food or lack of control over eating	6.20	4.20
Craving as a physiological state	4.34	3.89

BMI, body mass index; CES, compulsive eating scale; DASS-21, depression anxiety stress scales-21; EDDQ, eating disorder diagnostic questionnaire; FCQ-T, food craving questionnaire-trait; FCQ-S, food craving questionnaire-state; TFEQ-R18, three-factor eating questionnaire-R18.

### Experimental procedures

**FIGURE 1:**
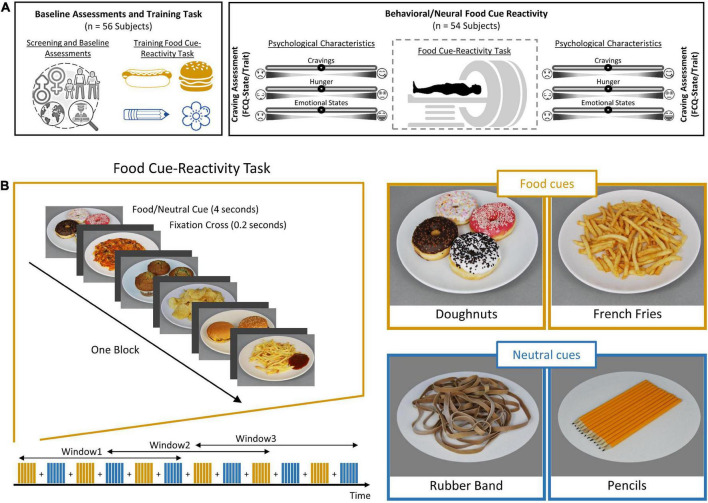
Outline of study design and food cue-reactivity task. **(A)** Experimental procedure. Individuals were first subjected to a cognitive functional screening. Each participant (*n* = 49) then underwent a food cue-reactivity training task. On the day of the experiment, participants completed the food craving questionnaire (FCQ)-state and FCQ-Trait questionnaires before undergoing an magnetic resonance (MR) scan with the food cue-reactivity task. Participants assessed their self-reported hunger, food craving, and emotional states before and after the MR scan. **(B)** Food cue-reactivity paradigm. In the food cue-reactivity task, participants saw 12 blocks of six pictures each (six blocks with food cues and six blocks with neutral cues). After each block, there was an inter-block interval that lasted between 8 and 12 s. Participants saw 72 stimuli throughout the course of 342 s.

After the screening procedure, each participant was scanned in a single fMRI session. On the screening session, participants completed a battery of baseline assessments {demographic questionnaire, Depression Anxiety Stress Scales-21 [DASS-21; (Sahebi et al., 2005)], Three-Factor Eating Questionnaire-R18 [TFEQ-R18; (Mostafavi et al., 2017)], Eating Disorder Diagnostic Scale [EDDS; (Stice et al., 2000)], Compulsive Eating Scale [CES; (Mostafavi et al., 2016)]}. This was followed by familiarization with the task during a training session outside the scanner, which lasted ∼3 min. On MRI scan day, participants arrived in the morning between 8:30 and 10:30 a.m., after not eating anything for at least 2 h. Participants were asked to complete Food Craving Questionnaire-State [FCQ-S; (Cepeda-Benito et al., 2000)] and the Food Craving Questionnaire-Trait [FCQ-T; (Kachooei and Ashrafi, 2016)]. After ratings of feelings of the current craving, hunger, prospective consumption, food control, and emotional states (anger, anxiety, awareness, drowsiness, happiness, and sadness) on a 0–100 visual analog scale (Figure 1A), participants began the food cue reactivity paradigm (in the MRI scanner). Participants assessed their feeling of food craving, prospective consumption, hunger, food control, and emotional states following the scans (Figure 1A).

### Food cue reactivity task

The task consisted of six blocks featuring a series of six food pictures each and six blocks featuring a series of six neutral pictures, used in our previous study (Ghobadi-Azbari et al., 2022). Each picture was presented for 4 s, and individual blocks were separated by 8–12 s intervals (Figure 1B). Participants saw 72 pictures throughout the course of 342 s. The order of presentation of stimulus was pseudo-randomized across subjects. The pseudo-randomized order was the same among subjects. The food and neutral cues were obtained from a publicly available and validated cue database (Charbonnier et al., 2016). Image presentation was controlled using the Psychtoolbox-3 software (Kleiner et al., 2007). We used a sliding window over the BOLD response to examine the temporal dynamics of brain activation, with a window size of 198 s and a window step size of 103 s, leading to the extraction of three overlapping windows. The overlapping nature of the sliding windows allows smoothing the transition between windows to have a higher signal-to-noise ratio, which increases the power of the LMEs and makes it possible to identify stable dynamic activate regions.

### MRI data acquisition

Functional images, field maps and an MPRAGE sequence were acquired on a Siemens Magnetom Prisma 3T scanner using a 20-channel head coil. For the functional MRI we used a T2*-weighted gradient echo planar (EPI) sequence with 43 transversal slices oriented parallel to the AC–PC line [whole-brain coverage, repetition time (TR) = 2,500 ms, echo time (TE) = 23 ms, flip angle (FA) = 70°, slice thickness 3.0 mm, FOV 192 mm, in-plane resolution 3 × 3 mm]. For the structural images we acquired a T1-weighted 3D MPRAGE sequence of 5 min 18 s (176 sagittal slices, slice thickness = 1 mm, TE = 3.45 ms, TR = 1,810 ms, FA = 7^°^, FOV = 256 × 256 mm). For the field map images we used a double-echo spoiled gradient echo sequence (gre_field_map; TR = 444 ms, TE = 5.19/7.65 ms, voxel size = 3 × 3 × 3 mm, FA = 60^°^).

### fMRI pre-processing

Functional MRI data were pre-processed with the AFNI software package (National Institute of Mental Health, Bethesda, MD)^1^. The first three functional scans were discarded to ensure steady-state magnetization. The pre-processing pipeline consisted of despiking, slice-time correction, realignment, co-registration, spatial normalization to the Montreal Neurological Institute (MNI) standardized space, and spatial smoothing with a 4 mm full-width at half-maximum Gaussian kernel. Times of repetition (TRs) with motion above 3 mm were censored.

### Functional MRI whole-brain contrast analysis

The preprocessed fMRI data were analyzed using a general linear model (GLM) created by modeling onset times for the food conditions and for the neutral conditions with a 25 s BLOCK function, convolved with a standard hemodynamic response function (HRF) to generate two regressors of interest. Six motion correction parameters were included from each subject as nuisance regressors into the first-level model. For each subject, the differential contrasts directly comparing the food to the neutral conditions were then entered into second-level mixed-effects models implemented using AFNI’s 3dMEMA. The whole-brain statistical group maps were corrected for multiple comparisons using a cluster-based approach. A Monte Carlo simulation using the AFNI’s 3dClustSim determined the cluster size threshold of 50 voxels corresponding to corrected statistical results with a voxel-wise bi-sided threshold of *p* < 0.0039 and with a threshold for whole brain multiple comparisons set at *p* < 0.05 using NN2 clustering, where clustered voxels can share faces and edges (Forman et al., 1995; Cox et al., 2017).

### Temporal dynamics of whole-brain activation

The effects of *Time* (T1, T2, T3), *Condition* (Food, Neutral), and *Time* × *Condition* interaction on the whole brain functional activation were analyzed with linear mixed effect (LME) using the function “3dLMEr” of the AFNI software package. *Condition* (Food, Neutral), *Time* (T1, T2, T3), and *Time* × *Condition* interaction were included as fixed-effects factors and Subjects as random effect. The resulting statistical group maps of main effect of *Time* are corrected for multiple comparisons using a cluster-based approach with a voxel-wise bi-sided *p*-value threshold of *p* < 0.0033 and a minimum cluster size of k = 40, which corresponds to a cluster-level alpha of *p* < 0.05 using NN2 clustering. The effects of Condition and time-by-condition interaction on the whole brain functional activation were analyzed with voxel-wise *p*-value thresholds of *p* < 0.05 and *p* < 0.01, respectively. The statistic values for main effects of *Time*, *Condition*, and *Time* × *Condition* interaction on the whole brain functional activation (generated by 3dLMEr) are represented in the output as chi-square with two degrees of freedom. The fixed number of DFs (i.e., 2) for the chi-square statistic, regardless of the specific situation, is adopted for convenience because of the varying DFs due to the Satterthwaite approximation.

### Temporal dynamics of regional activation

In order to examine the temporal dynamics of regional activation, we performed LME models (function *lmer* in R package *lme4*) for each subregion in the Brainnetome atlas with fixed effects of *Condition* (Food, Neutral) and *Time* in windows (T1, T2, T3), also adding an interaction term for the two effects (i.e., *Condition* and *Time*), and by-subject random intercepts. Also, the model included motion parameters as a fixed effect, which was defined as the mean Euclidean norm across the entire imaging run and included because of the potential for residual head motion effects after standard correction procedures. The model was “Beta∼Condition * Time + Motion + (1| Subject).” For each subject, mean beta weight values were estimated for all 246 ROIs in the Brainnetome atlas (BNA) (Fan et al., 2016). We then identified brain regions with significant time-by-condition interaction term. The *p*-values were not corrected in exploratory dynamic analysis of regional activation, and only uncorrected *p* < 0.05 values were used. Finally, we examined the temporal dynamics of significant regions using the BOLD signal change for the relevant conditions across the three windows. Information related to temporal dynamics of brain activation for block-wise analysis is presented in Supplementary material for Block-wise Temporal Dynamics.

### Group factor analysis

We used the group factor analysis (GFA) to explain relationships between groups of variables with a sparsity constraint (Klami et al., 2015). GFA uses a sparse Bayesian estimation to identify latent factors that either represent a robust relationship between groups or explain away group-specific variation. Four variable groups were defined: (1) brain activation measures; (2) behavioral measures; (3) demographic measures; and (4) baseline psychological measures. For brain activation measures, food minus neutral contrasts from 34 regions of interest [OFC (47o_left, 47o_right, A11l_left, A11l_right, A11m_left, A11m_right), vmPFC (A14m_left, A14m_right), ACC (A32p_left, A32p_right, A32sg_left, A32sg_right), caudate (dCa_left, dCa_right, vCa_left, vCa_right), putamen (dlPu_left, dlPu_right, vmPu_left, vmPu_right), globus pallidus (GP_left, GP_right), amygdala (lAmyg_left, lAmyg_right, mAmyg_left, mAmyg_right), nucleus accumbens (NAc_left, NAc_right), hippocampus (rHipp_left, rHipp_right), insula (vIa_left, vIa_right, vIg_left, vIg_right)] based on the results of meta-analyses were included as neural GFA group.

In total, the model included 34 regions of interest brain activation measures, 14 behavioral measures [changes in self-reported craving, FCQ-State subscales (*Lack of control, Desire, Positive reinforcement, Negative reinforcement, Physiological hunger*), and FCQ-Trait subscales (*Lack of control, Emotions, Guilt, Hunger, Thoughts*)], three demographic measures (Age, BMI, Education), and 10 self-report psychological measures [CES, DASS subscales (*Depression, Anxiety, Stress*), TEFQ subscales (*Hunger, Cognitive restraint, Emotional eating*), EDDQ subscales (*Body image, Overeating, Compensatory behaviors*)]. A form suited for GFA was achieved by z-normalizing the variables to have a zero mean and unit variance. To reduce the risk of identifying erroneous latent factors, GFA estimation was repeated 10 times to retain the robust latent factors constant across the sample chains.

### Individual habituation slopes

Following the group-level analysis’s identification of regions exhibiting dynamic activity, the relationship between the behavioral/clinical data and the individual habituation slopes for the regions with dynamic activity was examined (Plichta et al., 2014; Avery and Blackford, 2016). For each significant region of interest (ROI) in the *Time* × *Condition* interaction, we computed linear regression models separately for each subject using the function “lm” of the stats package in R programming language and entered the *Time* (with three windows) and *Condition* (food versus neutral), and their interaction as fixed effects. The model was “Beta∼Condition + Time + Condition: Time.” The slope of the interaction term, which represents the estimated beta coefficients of the “condition: time” term from these subject-level models, reflects the brain activity associated with one condition in the model in comparison to the other condition (habituation slopes). The time-by-condition interaction was considered to be the subject’s selective habituation to food-related cues, with negative values indicating a declining response to food-related cues as compared to the change in response to neutral cues.

## Results

### Behavioral effects of food cue reactivity

We investigated the behavioral effects of cue reactivity on craving, hunger, prospective consumption, and food control ratings. As expected, individuals demonstrated increased scores in craving [t(87.88) = –2.96, *P* = 0.004], hunger [t(53.30) = –2.69, *P* = 0.009], prospective consumption [t(86.99) = –3.19, *P* = 0.002], and decreased food control scores [t(56.67) = 2.12, *P* = 0.04] following food cue-reactivity task. Interestingly, we did not observe any statistically significant changes in anger [t(84.24) = 0.09, *P* = 0.925], awareness [t(91.75) = 0.98, *P* = 0.33], drowsiness [t(92.26) = 0.32, *P* = 0.75], sadness [t(92.95) = 0.49, *P* = 0.627], and happiness ratings [t(92.99) = –1.02, *P* = 0.311] following food cue-reactivity paradigm. Results revealed a significant decrease in anxiety state [t(84.67) = 2.46, *P* = 0.016]. Results are illustrated in Supplementary Figure 1.

### Time-invariant brain activation of food cue reactivity

**FIGURE 2:**
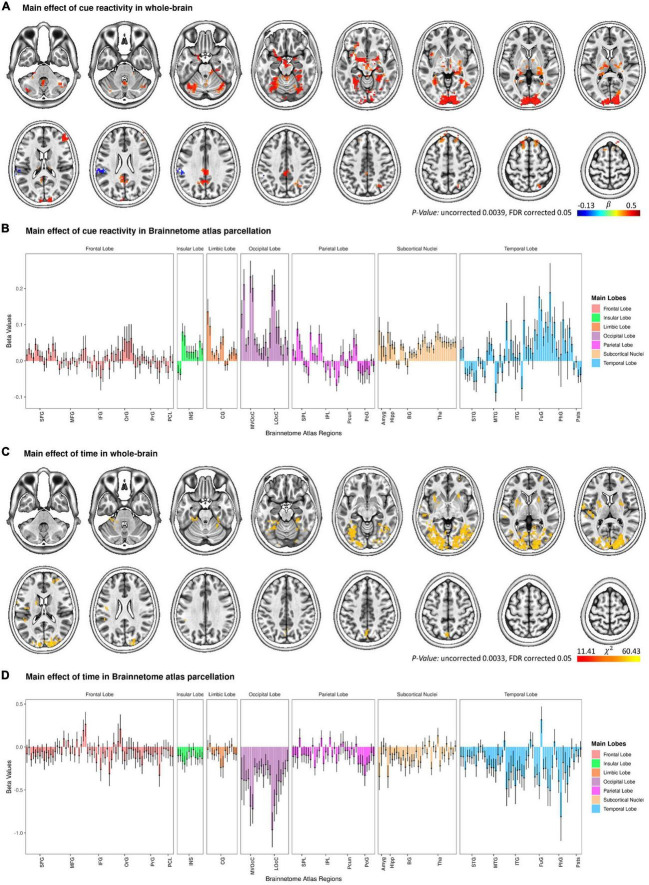
Change in functional brain activation during the functional magnetic resonance imaging (fMRI) food cue-reactivity task. Main effect of cue reactivity on the whole brain functional activation **(A)** and on Brainnetome atlas regions **(B)**. Main effect of *Time* on the whole brain functional activation **(C)** and on Brainnetome atlas regions **(D)**. Data in bar charts are represented as mean ± s.e.m. Tha, thalamus; BG, basal ganglia; Hipp, hippocampus; Amyg, amygdala; LOcC, lateral occipital cortex; MVOcC, medioventral occipital cortex; CG, cingulate gyrus; INS, insular gyrus; PoG, postcentral gyrus; Pcun, precuneus; IPL, inferior parietal lobule; SPL, superior parietal lobule; pSTS, posterior superior temporal sulcus; PhG, parahippocampal gyrus; FuG, fusiform gyrus; ITG, inferior temporal gyrus; MTG, middle temporal gyrus; STG, superior temporal gyrus; PCL, paracentral lobule; PrG, precentral gyrus; OrG, orbital gyrus; IFG, inferior frontal gyrus; MFG, middle frontal gyrus; SFG, superior frontal gyrus.

As a quality control to validate the activation pattern of food cue reactivity, a whole-brain GLM analysis was conducted with Food and Neutral as fixed regressors (see Figure 2A and Supplementary Table 1). As expected, the main effect of cue reactivity (contrast: food > neutral) was significant in several clusters. These clusters included regions in the medioventral/dorsal cingulate gyrus, medioventral fusiform gyrus, lateral superior frontal gyrus, ventral middle frontal gyrus, posterior orbitofrontal cortex, rostral superior/inferior parietal lobule, inferior semilunar lobule and the cerebellar tonsil, and occipital polar cortex. In addition, we also reported the brain activation results across the 246 subregions in the human Brainnetome Atlas (see Figure 2B).

### Dynamic brain activation of food cue-reactivity: Whole-brain analysis

#### Main effect of condition

The omnibus two-way (*Time* × *Condition*) LME model analysis revealed the main effect of *Condition* to be statistically significant in six clusters (with a voxel-wise *p*-value threshold of *p* < 0.05 and a minimum cluster size of k = 40; Supplementary Figure 2A and Supplementary Table 2). These clusters were located in the right superior parietal lobule, left medioventral fusiform gyrus, left cingulate gyrus, right parahippocampal gyrus, right precuneus, and right nucleus accumbens. In addition, we reported the results related to the main effect of *Condition* across the 246 regions of the Brainnetome atlas (see Supplementary Figure 2B).

#### Main effect of time

The LME model with *Condition* (Food, Neutral) and *Time* (T1, T2, T3) as fixed factors showed a significant main effect of *Time* in seven clusters (with a voxel-wise bi-sided *p*-value threshold of *p* < 0.0033 and a minimum cluster size of k = 40, which corresponds to a cluster-level alpha of *p* < 0.05 using NN2 clustering; Figure 2C and Supplementary Table 2). More interestingly, the two-way *Time* × *Condition* interaction was not significant for all clusters, reflecting attenuated responses to both categories of cues from T1 to T3. These clusters included regions in the occipital polar cortex, dorsal agranular insular gyrus, superior temporal gyrus, middle frontal gyrus, precuneus, ventromedial putamen, as well as in the posterior superior temporal sulcus. Figure 2D presents the main effect of *Time* across the 246 regions of the Brainnetome atlas.

#### Time × Condition interaction

At the whole-brain level, three clusters reached significance (with a voxel-wise *p*-value threshold of *p* < 0.01 and a minimum cluster size of k = 40). These clusters included regions in the precuneus and the occipital polar cortex, as well as in the medioventral fusiform gyrus (Supplementary Table 2).

### Dynamic brain activation of food cue-reactivity: Region of interest analysis

**FIGURE 3:**
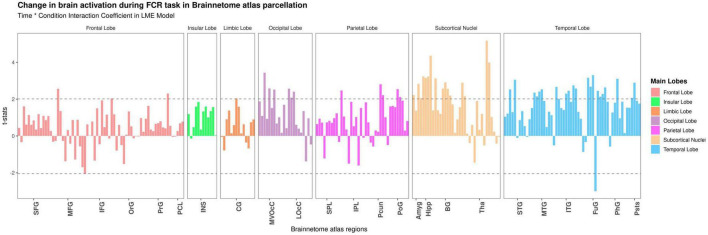
Detecting temporal dynamics on the Brainnetome atlas subregions. Time-by-condition interaction in the linear mixed effect (LME) model. Bars show the t-value of the time-by-condition interaction term in an LME (*Time × Condition* as fixed effect and subjects as a random effect) model for each subregion in the Brainnetome atlas (BNA). The horizontal dashed lines indicate brain regions with significant time-by-condition interaction term (*P* uncorrected <0.05). Tha, thalamus; BG, basal ganglia; Hipp, hippocampus; Amyg, amygdala; LOcC, lateral occipital cortex; MVOcC, medioventral occipital cortex; CG, cingulate gyrus; INS, insular gyrus; PoG, postcentral gyrus; Pcun, precuneus; IPL, inferior parietal lobule; SPL, superior parietal lobule; pSTS, posterior superior temporal sulcus; PhG, parahippocampal gyrus; FuG, fusiform gyrus; ITG, inferior temporal gyrus; MTG, middle temporal gyrus; STG, superior temporal gyrus; PCL, paracentral lobule; PrG, precentral gyrus; OrG, orbital gyrus; IFG, inferior frontal gyrus; MFG, middle frontal gyrus; SFG, superior frontal gyrus.

**FIGURE 4:**
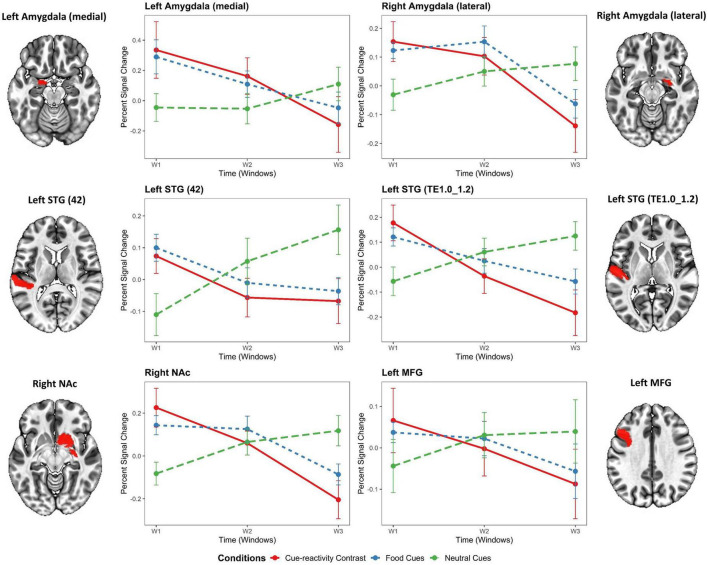
Temporal dynamics of regions of interest in the food cue-reactivity LME model. General linear model (GLM)-estimated percent signal change in response to food cue reactivity for each region of interest (ROI) with significant time-by-condition interactions in the LME model. Gray and orange labels denote the average main effect of food or neutral cue-exposure condition in the Brainnetome subregions or the result of food vs. neutral contrast. Error bars indicate s.e.m. across overweight and obese subjects (*N* = 49) at each temporal window. STG, superior temporal gyrus; NAc, nucleus accumbens; MFG, medial frontal gyrus.

At the ROI-based whole-brain level, several clusters reached significance (Figure 3). These clusters were located in the frontal lobe (IFJ, lateral MFG, lateral IFG, lateral PrG), limbic lobe (lateral cingulate gyrus), occipital lobe (rostral cuneus/caudal cuneus/rostral lingual MVOcC, OPC/MT + LOcC), parietal lobe (caudal IPL, dorsomedial parietooccipital sulcus, 3tonIa PoG, A2 PoG), temporal lobe [STG (TE1.0-TE1.2/42), caudal/rostral MTG, intermediate ventral/rostral/ventrolateral ITG, rostroventral/medioventral/lateroventral FuG, entorhinal cortex/rostral PhG, rostral Psts], and subcortical nuclei [medial/lateral amygdala, rostral/caudal hippocampus, ventral caudate, Nucleus Accumbens (NAc), dorsolateral/ventromedial putamen, occipital thalamus]. We selected six regions based on significant time-by-condition interactions in the LME models (Figure 3) and also based on regions involved in neural habituation in previous fMRI cue reactivity studies. Specifically, we explored the temporal dynamics of brain response to food and neutral stimuli for these six regions by independent LME models (i.e., LME models for each subregion in the Brainnetome atlas) (see Table 2). These two-way interactions were decomposed by *Condition*. Indeed, the food condition exhibited a declining trend in brain activation from T1 to T3 for all clusters (see Figure 4). In contrast, the neutral condition showed a progressive increase of brain activation from T1 to T3 (see Figure 4). For the six regions of interest with a dynamic involvement in food-related response reactivity, the value of contrast for food cue reactivity was estimated across the overlapping windows for further exploration, and the results are illustrated in Figure 4. All regions of interest with dynamic activity show habituation to food vs. neutral cues across time.

**TABLE 2:** Temporal dynamics of brain response to food and neutral stimuli for each region of interest (ROI) with significant time-by-condition interactions in the linear mixed effect (LME) model.

Fixed effects																					Random effects
	Motion					Time					Condition					*Time: Condition*					Subject
Regions in BNA	Beta	SE	95% interval low	95% interval high	*P*-value	Beta	SE	95% interval low	95% interval high	*P*-value	Beta	SE	95% interval low	95% interval high	*P*-value	Beta	SE	95% interval low	95% interval high	*P*-value	SD
Left amygdala (medial)	0.87	0.49	–0.09	1.83	0.078	–0.34	0.16	–0.65	–0.03	0.033	–0.35	0.22	–0.78	0.08	0.113	0.1	0.05	0.00	0.20	0.028	<0.001
Right amygdala (lateral)	0.47	0.25	–0.02	0.96	0.055	–0.03	0.08	–0.19	0.13	0.671	0.03	0.11	–0.19	0.25	0.761	0.26	0.13	0.01	0.51	0.045	<0.001
Left STG (42)	0.37	0.29	–0.20	0.94	0.202	–0.26	0.09	–0.44	–0.08	0.007	–0.24	0.13	–0.49	0.01	0.068	0.15	0.06	0.03	0.27	0.012	<0.001
Left STG (TE1.0_1.2)	0.35	0.25	–0.14	0.84	0.169	–0.11	0.08	–0.27	0.05	0.182	–0.17	0.11	–0.39	0.05	0.131	0.27	0.09	0.09	0.45	0.002	<0.001
Right NAc	0.11	0.28	–0.44	0.66	0.688	–0.07	0.09	–0.25	0.11	0.423	–0.06	0.13	–0.31	0.19	0.648	0.13	0.05	0.03	0.23	0.005	<0.001
Left MFG	0.14	0.28	–0.41	0.69	0.622	–0.09	0.09	–0.27	0.09	0.346	–0.04	0.12	–0.28	0.20	0.76	0.14	0.05	0.04	0.24	0.01	<0.001

SE, standard error; SD, standard deviation; STG, superior temporal gyrus; NAc, nucleus accumbens; MFG, medial frontal gyrus.

The independent LME models with *Condition* and *Time* (T1, T2, T3) as fixed factors showed significant time-by-condition interactions in the left medial amygdala [t(289) = 2.21, β = 0.1, *P* = 0.028], right lateral amygdala [t(289) = 2.01, β = 0.26, *P* = 0.045], left STG [42 Area: t(289) = 2.53, β = 0.15, *P* = 0.012; TE1.0_TE1.2 Area: t(289) = 3.13, β = 0.27, *P* = 0.002], right NAc [t(289) = 2.81, β = 0.13, *P* = 0.005], and left MFG [t(289) = 2.58, β = 0.14, *P* = 0.01]. In addition, the LME models exhibited a significant main effect for time in the left medial amygdala [t(289) = –2.14, β = –0.34, *P* = 0.033] and left STG [42 Area: t(289) = –2.71, β = –0.26, *P* = 0.007] but no significant effect in the right lateral amygdala [t(289) = –0.43, β = –0.03, *P* = 0.671], left STG [TE1.0_TE1.2 Area: t(289) = –1.34, β = –0.11, *P* = 0.182], right NAc [t(289) = –0.80, β = –0.07, *P* = 0.423], and left MFG [t(289) = –0.94, β = –0.09, *P* = 0.346]. Interestingly, the LME models revealed no significant main effect for *Condition* in the left medial amygdala [t(289) = –1.59, β = –0.35, *P* = 0.113], right lateral amygdala [t(289) = 0.30, β = 0.03, *P* = 0.761], left STG [42 Area: t(289) = –1.83, β = –0.24, *P* = 0.068; TE1.0_TE1.2 Area: t(289) = –1.51, β = –0.17, *P* = 0.131], right NAc [t(289) = –0.46, β = –0.06, *P* = 0.648], and left MFG [t(289) = –0.31, β = –0.04, *P* = 0.760]. As expected, no significant effect of *Motion* was found for all regions (see Table 2).

### Brain-behavior relationships

#### Group factor analysis

**FIGURE 5:**
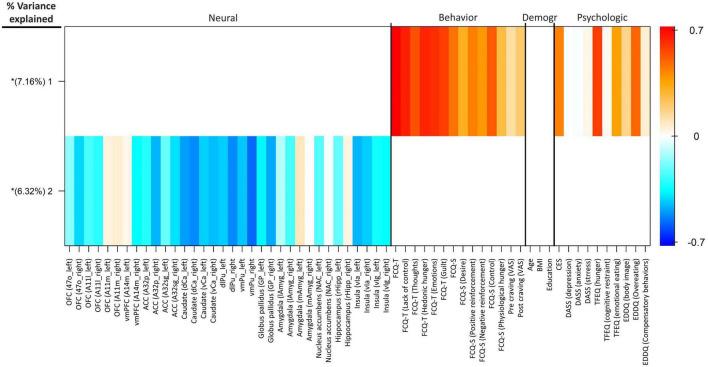
Group factor analysis. Heatmap colors represent the weight of each variable loading. The x-axis shows the variables included in each group. Extracted robust group factors and the percentage of variance explained by each are shown on the y-axis. Asterisks show group factors that contained at least one loading weight whose 95% credible interval did not contain 0. OFC, orbitofrontal cortex; vmPFC, ventromedial prefrontal cortex; ACC, anterior cingulate cortex; dCa, dorsal caudate; vCa, ventral caudate; dlPu, dorsolateral putamen; vmPu, ventromedial putamen; GP, globus pallidus; lAmyg, lateral amygdala; mAmyg, medial amygdala; NAc, nucleus accumbens; rHipp, rostral hippocampus; vIa, ventral agranular insular; vIg, ventral granular insular; FCQ-S, food craving questionnaire-state; FCQ-T, food craving questionnaire-trait; VAS, visual analog scale; BMI, body mass index; CES, compulsive eating scale; DASS, depression anxiety stress scales; TFEQ, three-factor eating questionnaire; EDDQ, eating disorder diagnostic questionnaire; Demogr, demographic.

By using group factor analysis, two robust latent variables were identified, which together explain 13.48% of the variance across variable groups (see Figure 5). There were no robust cross-unit latent factors found between the neural group with behavioral, demographic, and self-report psychological groups. In other words, the GFA failed to demonstrate any coherence in the latent variable space between the neural group with the behavioral, demographic, and self-report psychological groups. In contrast, a robust latent factor that loaded across the behavioral and self-report psychological groups of analysis was found using the GFA. The mean-variance explained for the groups of behavioral and self-report psychological variables, however, was 25.08 and 8.45%, respectively. As a result, while this latent factor had loadings at both the behavioral and self-report psychological levels of analysis, the behavioral variables accounted for almost all of the variance explained.

#### Individual habituation slopes

We tested for bivariate correlations between self-report psychological measures [DASS, DASS subscales (Depression, Anxiety, Stress), CES], behavioral measures [FCQ-Trait, FCQ-Trait subscales (Lack of control, Emotions, Guilt, Hunger, Thoughts), FCQ-State, FCQ-State subscales (Lack of control, Desire, Positive reinforcement, Negative reinforcement, Physiological hunger), craving self-reports] with individual habituation slopes within the six regions of interest. Here, the individual habituation slope in the left medial amygdala was correlated with DASS subscales [Depression (R = –0.12; *P* = 0.01), Anxiety (R = –0.09; *P* = 0.012), and Stress (R = –0.13; *P* = 0.007)] and overall DASS (R = –0.13; *P* = 0.008). The individual habituation slope in the right lateral amygdala was correlated with CES (R = –0.11; *P* = 0.031), Emotions subscale of the FCQ-T (R = –0.23; *P* = 0.020), and self-reported craving before scanning (R = 0.28; *P* = 0.013). The individual habituation slope in the left STG (42) was positively and significantly correlated with self-reported craving before scanning (R = –0.27; *P* = 0.014). Finally, the individual habituation slope in the left MFG was negatively and significantly related to FCQ-S subscales [Desire (R = –0.10; *P* = 0.024) and Physiological hunger (R = –0.08; *P* = 0.036)] and craving self-reports before (R = –0.18; *P* = 0.007) and after (R = –0.15; *P* = 0.024) scanning. Supplementary Figure 3 shows the relationship between the self-report psychological measures and behavioral measures with individual habituation slopes within the six regions of interest.

## Discussion

The present study examined the temporal dynamics of regional activation on the performance of food cue reactivity in overweight and obese people. The primary novel finding of this study involves the demonstration of habituation of activity of the amygdala, superior temporal gyrus, nucleus accumbens, and medial frontal gyrus to presentations of food vs. neutral cues across time in overweight and obese people. We found that none of the brain regions with dynamic activity shows an increased response to food cues over time (sensitization) in overweight and obese people. Notably, dynamic amygdala activity with a similar downward slope over time has been observed in the recent cue-reactivity studies (Murphy et al., 2018; Ekhtiari et al., 2021; Jafakesh et al., 2022). For example, a study also similarly reported dynamic cue-reactivity in the STG, but they observed an initially escalating and subsequently decreasing activation whereas we observed a consistent habituation response (Ekhtiari et al., 2021). Meanwhile, our findings show an initially escalating and subsequently decreasing response to food cues (not food vs. neutral) in the right amygdala and right NAc regions. Our findings suggest generalized habituation to food vs. neutral stimuli within regions with time-varying activity.

Habituation of the neural response to repeated exposure to salient visual stimuli is well-documented, both at the animal and human level. Neuroimaging studies consistently report a distinct engagement of the regions supporting salience and rewards processes in the habituation response to the salient cues. However, the pattern of these habituation effects varies, depending on the types of stimuli. Accordingly, our findings reveal a time-by-condition interaction, indicating a temporally dynamic response to the repeated presentation of food cues. The anatomic location of the clusters with significant time-by-condition interaction falls within the medial or lateral amygdala, superior temporal gyrus, nucleus accumbens, and medial frontal gyrus, implicated in the salience and rewards processing. The present study adds to earlier findings that repeatedly viewing salient visual stimuli leads to habituation of limbic subcortical (e.g., amygdala, hippocampus) and cortical (DLFPC, precentral gyrus) areas.

Neuroimaging studies strongly support a central role of superior temporal gyrus (STG) in food cue reactivity (Nummenmaa et al., 2012; Drummen et al., 2018; Bach et al., 2021). Our whole-brain contrast analysis (contrast: food > neutral) reveal significant decreases in the average BOLD signal (deactivation) in the STG during food cue-reactivity task. Interestingly, although no significant main effect of *Condition* was observed in the STG during the paradigm of food cue reactivity using LME analysis (Table 2), however, we observed a significant time-by-condition interaction in the STG, which points to a temporal dynamic involvement in food-related response reactivity. This discovery might indeed be explained by the STG’s function in the visuotemporal attentional processing of environmental cues (Li et al., 2012). Previous research found that damage to the STG leads to a prolonged and more severe impairment in visuotemporal attention (Shapiro et al., 2002). In line with our expectations, food cues may cause the STG to respond quickly to attentionally salient stimuli. However, it is interesting to note that, the attentional saliency tends to be decrease quickly for food cues, and in general, there is no stronger activation for food cues in the STG compared to neutral stimuli.

By suppressing the association between the conditioned stimulus and the unconditioned stimulus, food cue exposure treatment, a therapeutic strategy based on the Pavlovian conditioning paradigm, aims to extinguish cravings and prevent future relapses (van den Akker et al., 2014). However, the outcome of the cue-exposure effects varies, depending on sufficient reductions, or habituation, of anxiety (or cravings) throughout therapy (Agarwal et al., 2021). Inhibitory learning models of extinction provide strong anchors for understanding the mechanisms of cue exposure therapy in psychiatric disorders (Craske et al., 2014; Schyns et al., 2020). For example, the famous revised model of inhibitory learning elaborated by Craske et al. (2014) provides a mechanistic framework based on neurocognitive resources to strengthen inhibitory learning between a fear-associated stimulus and the co-occurrence of an aversive outcome. Brain areas associated with inhibitory control include the caudate, dorsal anterior cingulate cortex, dorsolateral prefrontal cortex, globus pallidus, parietal posterior cortex, presupplementary motor area, and ventrolateral prefrontal cortex (Agarwal et al., 2021). On exposure to visual food cues, increased inhibitory control activation in the dorsolateral prefrontal cortex was related to a stronger reduction of food craving in obese people (Demos McDermott et al., 2019). Moreover, successfully avoiding from consuming high-calorie foods is related to decreased cue reactivity, which in turn makes it simpler to resist enticing foods and encourage weight reduction (Janssen et al., 2017). Furthermore, stimulating the medial prefrontal cortex with deep repetitive transcranial magnetic stimulation in a randomized double-blind sham-controlled trial enhanced the effectiveness of cue exposure therapy (Ceccanti et al., 2015). In order to reduce the affective response in individuals with psychiatric disorders, fMRI neurofeedback research are also focusing on habituation in response to affective stimuli, including brain areas such as the medial prefrontal cortex, amygdala, and ventral striatum (Young et al., 2014; Nicholson et al., 2017; Zotev et al., 2018). Similarly, we found in our research that, out of several brain regions that were activated by exposure to the food cues, the amygdala, PFC, NAc, and STG demonstrated significant habituation in response to repeated exposure to food cues within the temporal window of our fMRI cue-reactivity task. These findings can support the potential role of conventional cue exposure therapies in habituation and learning extinction across a select number of the neurocognitive processes implicated in food cravings.

### Limitations

This study is not without limitations. First, because the order was not pseudo-randomized, the participants could predict the type of the next block, which might have an impact on the outcomes. Second, although we didn’t distinguish between high-calorie and low-calorie food images in our cue presentation, research have shown that high-calorie and low-calorie food images may have distinct effects on the brain (Stoeckel et al., 2009; Schulte et al., 2019). Third, all participants were exposed to the same stimuli, despite evidence that different persons have varied interests and hence respond differently to the same cues. Tailoring should be done to address this issue (Franssen et al., 2020). Fourth, due to power problems, we took the blocks together without separating them based on whether they were food- or neutral-related. To further examine these findings, larger research with more task durations is required (Luan Phan et al., 2003).

## Conclusion

In summary, the present results demonstrate for the first time that the regions of the rewards and inhibitory processing show fluctuations to repeated exposure to food visual stimuli consistent with habituation. This study also underscores the importance of examining the temporal dynamics of brain activation in fMRI design and analysis in studies of food cue reactivity, thereby suggesting pathways in biomarker development and cue-desensitization interventions.

## Data availability statement

The raw data supporting the conclusions of this article will be made available by the authors, without undue reservation.

## Ethics statement

The studies involving human participants were reviewed and approved by Iran University of Medical Science (IR.IUMS.REC.1396.0459). The patients/participants provided their written informed consent to participate in this study.

## Author contributions

PG-A and HE designed the research. PG-A performed the research, analyzed the data, and wrote the manuscript. HE and RM reviewed and edited the manuscript and supervised the manuscript. All authors approved the final manuscript.

## Footnotes

1
https://afni.nimh.nih.gov
