# Supplementary material for: Habituation or sensitization of brain response to food cues: Temporal dynamic analysis in an functional magnetic resonance imaging study
Source: Front Hum Neurosci. 2023 Feb 17;17:1076711. doi: 10.3389/fnhum.2023.1076711 (PMC9983367; doi:10.3389/fnhum.2023.1076711)
Supplement: Supplementary file 1 [file Data_Sheet_1.docx]

**Supplementary Material**

Title: **Habituation or Sensitization of Brain Response to Food Cues: Temporal Dynamic Analysis in an fMRI Study**

**Authors:** Peyman Ghobadi-Azbari^1^, Rasoul Mahdavifar Khayati^1*^, Hamed Ekhtiari^2^

1. Department of Biomedical Engineering, Shahed University, Tehran, Iran

2. Department of Psychiatry, University of Minnesota, Minnesota, USA

***Corresponding Author:**

email: mahdavifar@shahed.ac.ir

**Supplementary Material for Block-wise Temporal Dynamics**

**Supplementary Methods and Material** **for Block-wise Temporal Dynamics**

**Food cue reactivity task**

In the fMRI food cue-reactivity task, participants were presented with blocks of cues that are either food-related or neutral. Each block contains six images presented for four seconds each, with an inter-stimulus interval of 200 ms between cues so that an entire block lasts 25 seconds. Individual blocks were separated by 8‐12 second intervals. A total of 12 blocks were presented, six of each condition. Participants saw 72 pictures throughout the course of 342 seconds (Figure A).

**
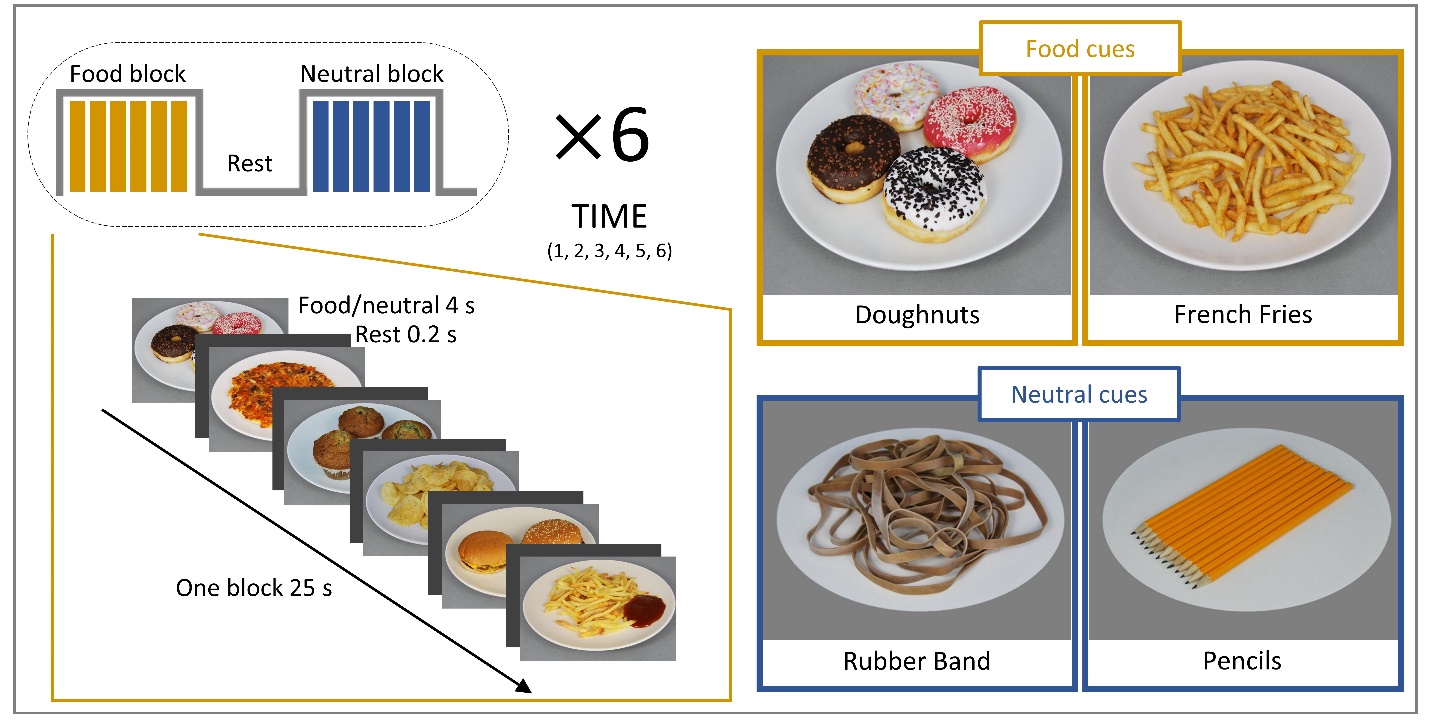
**

**Figure A.** **fMRI food cue-reactivity task structure and timings.** There are 12 blocks of 6 pictures each (6 blocks with food cues and 6 blocks with neutral cues). Each picture is presented for 4 seconds with 0.2 seconds inter-stimulus interval. After each block, there was an inter-block interval that lasted between 8 and 12 seconds. Participants saw 72 stimuli throughout the course of 342 seconds.

**Temporal Dynamics of Regional Activation**

In order to examine the temporal dynamics of regional activation, we performed LME models (function *lmer* in R package *lme4*) for each subregion in the Brainnetome atlas with fixed effects of *Condition* (Food, Neutral), *Time* (T1, T2, T3, T4, T5, T6), and *Time* × *Condition* interaction and *Subjects* as random effect. For each subject, mean beta weight values were estimated for all 246 ROIs in the Brainnetome atlas (BNA). We then identified brain regions with significant time-by-condition interaction term. Additionally, we repeated all analyzes taking into account *Age* as a control covariate and results were nearly identical. Finally, we examined the temporal dynamics of significant regions using the BOLD signal change for the relevant conditions across the six windows.

As a less robust, complementary test to investigate the temporal dynamics of regional activation, we used ANOVA (function *anova* in base R) for each subregion in the Brainnetome atlas. *Condition* (Food, Neutral), *Time* (T1, T2, T3, T4, T5, T6), and *Time* × *Condition* interaction were included as fixed-effects factors. For each subject, mean beta weight values were estimated for all 246 ROIs in the BNA.

**Individual Habituation Slopes**

Individual-level dynamic responses (habituation slopes) were estimated for each ROI exhibiting a significant time-by-condition interaction (Plichta et al., 2014; Avery and Blackford, 2016). Slopes were computed by fitting linear regression models separately for each subject with “Beta ~ Condition + Time + Condition: Time” using the function “lm” of the stats package in R programming language. *Condition* (Food, Neutral), *Time* (T1, T2, T3, T4, T5, T6), and *Time* × *Condition* interaction were included entered as fixed-effects factors. The slope of the interaction term, which represents the estimated beta coefficients of the "Condition: Time" term from the subject-level models, reflects the brain activity associated with one condition in the model in comparison to the other condition (habituation slopes). The time-by-condition interaction was considered to be the subject's selective habituation to food-related cues, with negative values indicating a declining response to food-related cues as compared to the change in response to neutral cues. Finally, we examined the relationship between the individual habituation slopes of significant regions with behavioral and clinical data.

**Supplementary Results for Block-wise Temporal Dynamics**

**Temporal Dynamics of Regional Activation**

At the regional level, several regions reached significance (Figure B). These regions were located in the subcortical nuclei (left and right ventral caudate (vCa), right nucleus accumbens (NAc), right ventromedial putamen (vmPu), and left sensory thalamus (Stha)). Results related to the temporal dynamics of brain response during food cue reactivity for significant *Time* × *Condition* interaction regions by independent LME models are presented in Table A.


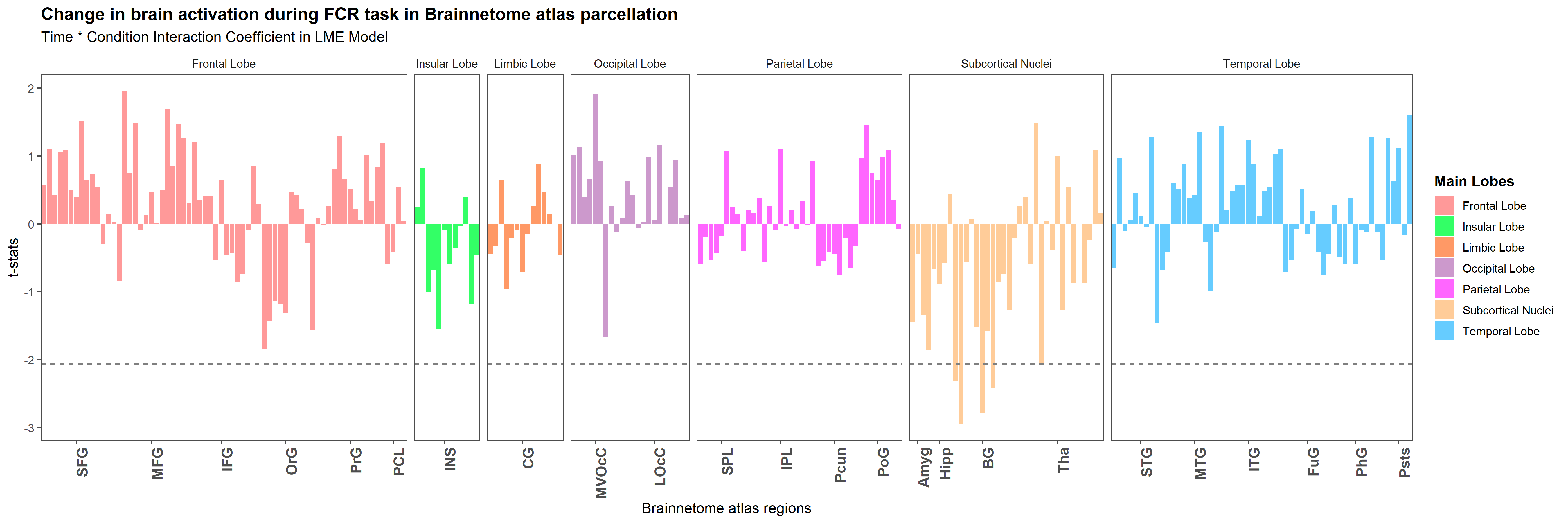
 **Figure B.** **Condition-by-time interactions in the LME models**. Bars show the t-value of the time-by-condition interaction term in an LME (*Condition x Time* as fixed effect and Subjects as a random effect) model for each subregion in the BNA. The horizontal dashed lines indicate brain regions with significant time-by-condition interaction term (P uncorrected < 0.05). **Abbreviations:** Tha, thalamus; BG, basal ganglia; Hipp, hippocampus; Amyg, amygdala; LOcC, lateral occipital cortex; MVOcC, medioventral occipital cortex; CG, cingulate gyrus; INS, insular gyrus; PoG, postcentral gyrus; Pcun, precuneus; IPL, inferior parietal lobule; SPL, superior parietal lobule; pSTS, posterior superior temporal sulcus; PhG, parahippocampal gyrus; FuG, fusiform gyrus; ITG, inferior temporal gyrus; MTG, middle temporal gyrus; STG, superior temporal gyrus; PCL, paracentral lobule; PrG, precentral gyrus; OrG: orbital gyrus; IFG: inferior frontal gyrus; MFG, middle frontal gyrus; SFG, superior frontal gyrus.

The independent LME models with *Condition* (Food, Neutral) and *Time* (T1, T2, T3, T4, T5, T6) as fixed factors showed significant time-by-condition interactions in the left vCa (β = -0.071, P = 0.021), right vCa (β = -0.059, P = 0.003), right NAc (β = -0.073, P = 0.006), right vmPu (β = -0.049, P = 0.016), and left Stha (β = -0.042, P = 0.040). In addition, the LME models exhibited a significant main effect for *Time* in the left vCa (β = 0.111, P = 0.024) but no significant effect in the right vCa (β = 0.056, P = 0.079), right NAc (β = 0.071, P = 0.087), right vmPu (β = 0.03, P = 0.353), and left Stha (β = 0.031, P = 0.334). Additionally, the LME models revealed a significant main effect for *Condition* in the right vCa (β = 0.155, P = 0.047) and right NAc (β = 0.204, P =0.046) but no significant effect in the left vCa (β = 0.171, P = 156), right vmPu (β = 0.139, P = 0.075), and left Stha (β = 0.078, P = 0.327). Interestingly, the LME models revealed no significant main effect of *Age* for regions of interest (left and right vCa, right NAc, right vmPu, left Stha) (see Table A).

For the regions of interest with a dynamic involvement in food cue reactivity (i.e., right vCa, right NAc, right vmPu, left Stha), the BOLD signal changes were estimated across the six windows for further exploration, and the results are illustrated in Figure C.

The ANOVA analyses revealed the effect of time-by-condition interaction to be statistically significant at the regional level (Figure D). These regions were located in the subcortical nuclei (left and right vCa, right NAc, and right vmPu). Results related to the temporal dynamics of brain response during food cue reactivity for significant time-by-condition interaction regions by ANOVA are presented in Table B. The ANOVA analysis with *Condition* (Food, Neutral) and *Time* (T1, T2, T3, T4, T5, T6) as fixed factors showed significant time-by-condition interactions in the left vCa (F(1,572) = 5.343, P = 0.021), right vCa (F(1,572) = 8.206, P = 0.004), right NAc (F(1,572) = 7.222, P = 0.007), and right vmPu (F(1,572) = 5.373, P = 0.021). In addition, the ANOVA analyses exhibited the significant main effect for *Time* in the right vCa (F(1,572) = 10.125, P = 0.002), right NAc (F(1,572) = 7.992, P = 0.005), and right vmPu (F(1,572) = 17.103, P < 0.001) but no significant effect in the left vCa (F(1,572) = 0.06, P = 0.806). There were no significant effect of *Condition* in the left vCa (F(1,572) = 2.232, P = 0.136), right vCa (F(1,572) = 2.092, P = 0.149), right NAc (F(1,572) = 1.182, P = 0.277), and right vmPu (F(1,572) = 0.729, P = 0.394).

| **Table A.** Temporal dynamics of brain response to food and neutral stimuli for each ROI with significant time-by-condition interactions in the LME model. | | | | | | | | | | | | | | | | | | | | | | | | | | |
| --- | --- | --- | --- | --- | --- | --- | --- | --- | --- | --- | --- | --- | --- | --- | --- | --- | --- | --- | --- | --- | --- | --- | --- | --- | --- | --- |
|  |  | **Fixed Effects** | | | | | | | | | | | | | | | | | | | | | | |  | **Random Effects** |
|  |  | **Age** | | | | |  | **Time** | | | | |  | **Condition** | | | | |  | **Time:Condition** | | | | |  | **Subject** |
| **Regions in BNA** |  | **Beta** | **SE** | **95% Interval Low** | **95% Interval High** | **P-value** |  | **Beta** | **SE** | **95% Interval Low** | **95% Interval High** | **P-value** |  | **Beta** | **SE** | **95% Interval Low** | **95% Interval High** | **P-value** |  | **Beta** | **SE** | **95% Interval Low** | **95% Interval High** | **P-value** |  | **SD** |
| Left vCa |  | -0.002 | 0.003 | -0.008 | 0.004 | 0.538 |  | 0.111 | 0.049 | 0.015 | 0.207 | 0.024 |  | 0.171 | 0.121 | -0.066 | 0.408 | 0.156 |  | -0.071 | 0.031 | -0.132 | -0.010 | 0.021 |  | < 0.001 |
| Right vCa |  | -0.002 | 0.002 | -0.006 | 0.002 | 0.444 |  | 0.056 | 0.032 | -0.007 | 0.119 | 0.079 |  | 0.155 | 0.078 | 0.002 | 0.308 | 0.047 |  | -0.059 | 0.02 | -0.098 | -0.020 | 0.003 |  | 0.099 |
| Right NAc |  | -0.001 | 0.003 | -0.007 | 0.005 | 0.734 |  | 0.071 | 0.041 | -0.009 | 0.151 | 0.087 |  | 0.204 | 0.102 | 0.004 | 0.404 | 0.046 |  | -0.073 | 0.026 | -0.124 | -0.022 | 0.006 |  | 0.145 |
| Right vmPu |  | -0.002 | 0.002 | -0.006 | 0.002 | 0.417 |  | 0.03 | 0.032 | -0.033 | 0.093 | 0.353 |  | 0.139 | 0.078 | -0.014 | 0.292 | 0.075 |  | -0.049 | 0.02 | -0.088 | -0.010 | 0.016 |  | 0.125 |
| Left Stha |  | -0.003 | 0.003 | -0.009 | 0.003 | 0.377 |  | 0.031 | 0.032 | -0.032 | 0.094 | 0.334 |  | 0.078 | 0.079 | -0.077 | 0.233 | 0.327 |  | -0.042 | 0.02 | -0.081 | -0.003 | 0.04 |  | 0.202 |
| **Abbreviations:** BNA, Brainnetome atlas; SE, standard error; SD, standard deviation; vCa, ventral caudate; NAc, nucleus accumbens; vmPu, ventromedial putamen; Stha, sensory thalamus. | | | | | | | | | | | | | | | | | | | | | | | | | | |

**
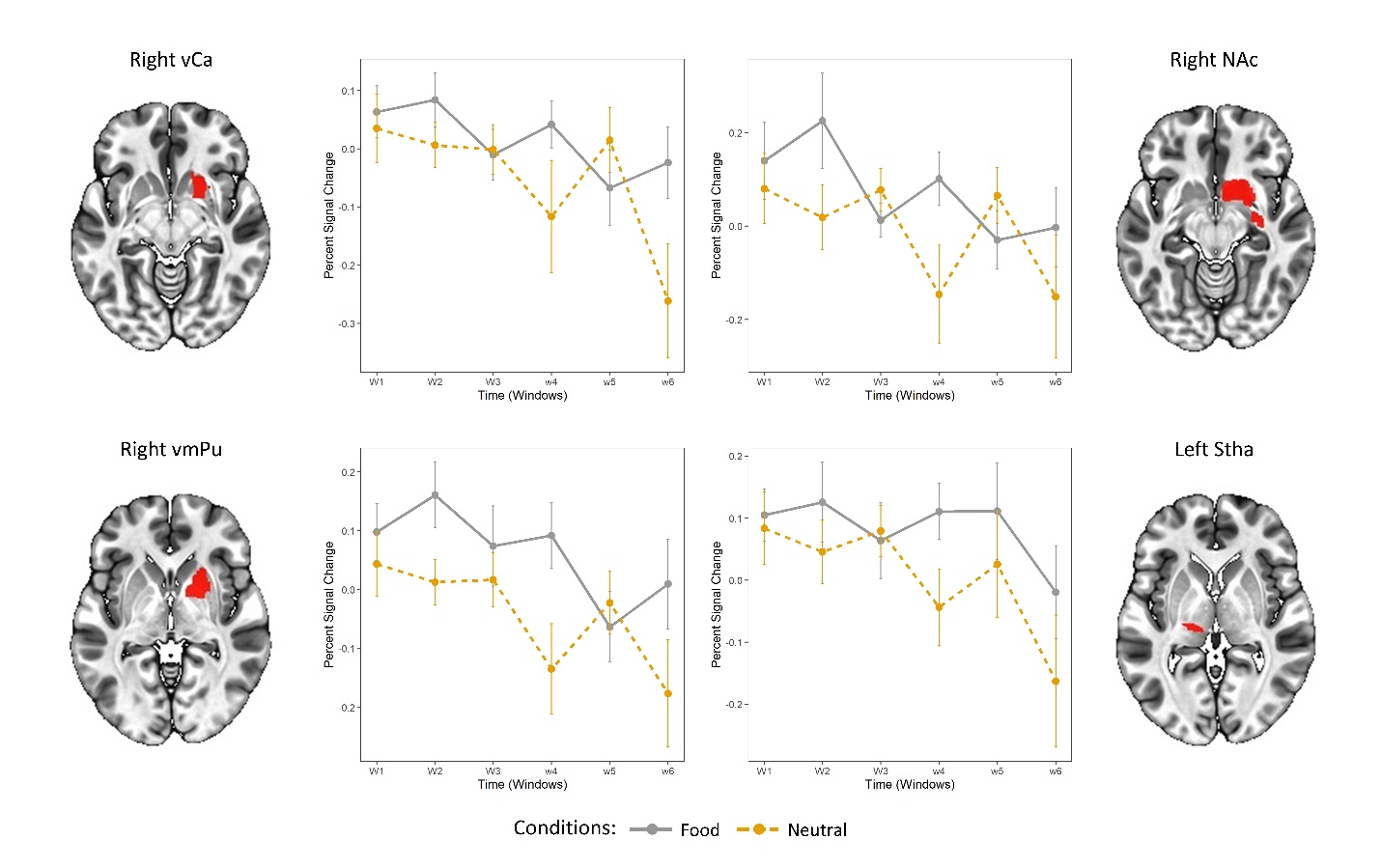
**

**Figure C. Temporal dynamics of brain response for food cue reactivity.** The BOLD percent signal change in response to food or neutral stimuli for each ROI with significant time-by-condition interactions in the LME model. Gray and orange labels denote the average main effect of food and neutral condition in the Brainnetome subregions, respectively. Error bars indicate s.e.m. across subjects (N = 49) at each window. **Abbreviations:** vCa, ventral caudate; NAc, nucleus accumbens; vmPu, ventromedial putamen; Stha, sensory thalamus.

**
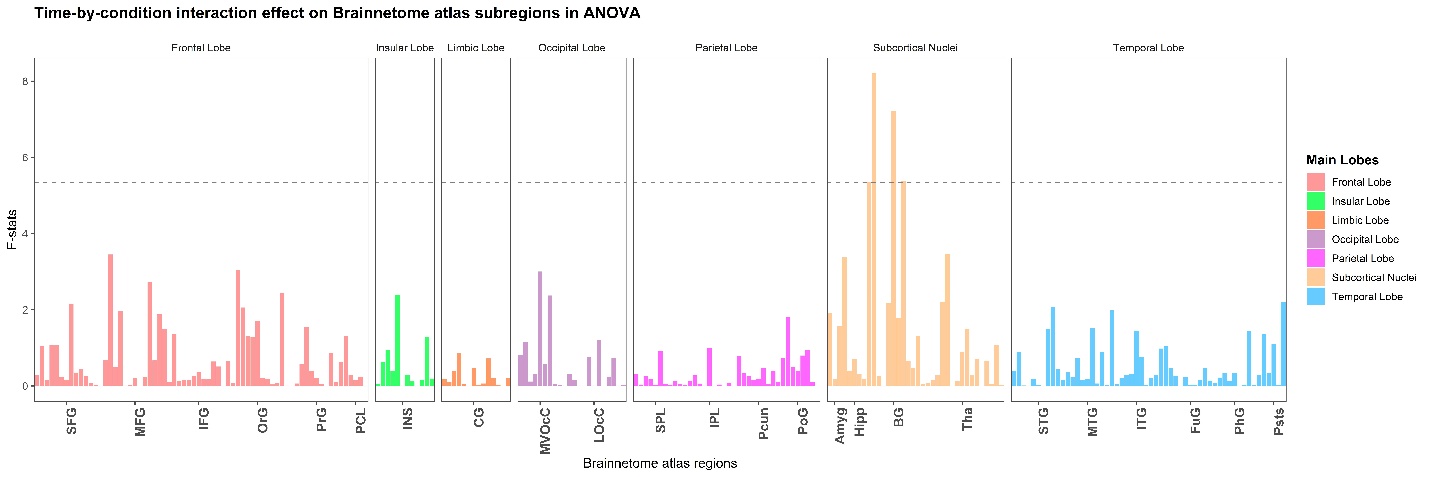
Figure D.** **Time-by-condition interactions in the ANOVA**. Bars show the F-value of the time-by-condition interaction term in an ANOVA for each subregion in the BNA. The horizontal dashed lines indicate brain regions with significant time-by-condition interaction term (P uncorrected < 0.05). **Abbreviations:** Tha, thalamus; BG, basal ganglia; Hipp, hippocampus; Amyg, amygdala; LOcC, lateral occipital cortex; MVOcC, medioventral occipital cortex; CG, cingulate gyrus; INS, insular gyrus; PoG, postcentral gyrus; Pcun, precuneus; IPL, inferior parietal lobule; SPL, superior parietal lobule; pSTS, posterior superior temporal sulcus; PhG, parahippocampal gyrus; FuG, fusiform gyrus; ITG, inferior temporal gyrus; MTG, middle temporal gyrus; STG, superior temporal gyrus; PCL, paracentral lobule; PrG, precentral gyrus; OrG: orbital gyrus; IFG: inferior frontal gyrus; MFG, middle frontal gyrus; SFG, superior frontal gyrus.

| **Table B.** Temporal dynamics of brain response to food and neutral stimuli for each ROI with significant time-by-condition interactions in the ANOVA. | | | | | | | | | | | | | |
| --- | --- | --- | --- | --- | --- | --- | --- | --- | --- | --- | --- | --- | --- |
|  | **Time** | | |  | **Condition** | | |  | | **Time:Condition** | | | |
| **Regions in BNA** | **Mean sq** | **F-value** | **P-value** |  | **Mean sq** | **F-value** | **P-value** |  | **Mean sq** | | **F-value** | **P-value** |  |
| Left vCa | 0.024 | 0.060 | 0.806 |  | 0.897 | 2.232 | 0.136 |  | 2.146 | | 5.343 | 0.021 |  |
| Right vCa | 1.794 | 10.125 | 0.002 |  | 0.371 | 2.092 | 0.149 |  | 1.454 | | 8.206 | 0.004 |  |
| Right NAc | 2.446 | 7.992 | 0.005 |  | 0.362 | 1.182 | 0.277 |  | 2.210 | | 7.222 | 0.007 |  |
| Right vmPu | 3.151 | 17.103 | <0.001 |  | 0.134 | 0.729 | 0.394 |  | 0.990 | | 5.373 | 0.021 |  |
| **Abbreviations:** BNA, Brainnetome atlas; Mean sq, mean of the sum of squares; vCa, ventral caudate; NAc, nucleus accumbens; vmPu, ventromedial putamen. | | | | | | | | | | | | | |

**Individual Habituation Slopes**

Table C shows the bivariate correlations between the self-report psychological measures (DASS, DASS subscales (Depression, Anxiety, Stress), CES), behavioral measures (FCQ-Trait, FCQ-Trait subscales (Lack of control, Emotions, Guilt, Hunger, Thoughts), FCQ-State, FCQ-State subscales (Lack of control, Desire, Positive reinforcement, Negative reinforcement, Physiological hunger), craving self-reports) with individual habituation slopes within the regions of interest for the six-window analysis. Here, the individual habituation slope in the left ventral caudate was strongly correlated with overall FCQ-T (R = -0.225; P < 0.001), Hedonic hunger subscale of the FCQ-T (R = -0.328; P < 0.001), and Positive reinforcement subscale of the FCQ-S (R = -0.218; P < 0.001). The individual habituation slope in the right ventral caudate was strongly correlated with Hedonic hunger subscale of the FCQ-T (R = -0.216; P < 0.001), FCQ-S subscales (Positive reinforcement (R = -0.245; P < 0.001) and Control (R = -0.235; P < 0.001)), and self-reported craving after scanning (R = -0.219; P = 0.014). The individual habituation slope in the right nucleus accumbens was negatively and significantly correlated with Hedonic hunger subscale of the FCQ-T (R = -0.234; P < 0.001), Control subscale of the FCQ-S (R = -0.202; P < 0.001), and self-reported craving after scanning (R = -0.275; P = 0.007). The individual habituation slope in the right ventromedial putamen was correlated with CES (R = -0.213; P < 0.001), FCQ-T subscales (Lack of control (R = -0.241; P < 0.001) and Hedonic hunger (R = -0.318; P < 0.001)), overall FCQ-T (R = -0.251; P < 0.001), FCQ-S subscales (Desire (R = -0.202; P < 0.001), Positive reinforcement (R = -0.365; P < 0.001), Negative reinforcement (R = -0.226; P < 0.001), Control (R = -0.360; P < 0.001)), overall FCQ-S (R = -0.3; P < 0.001), and self-reported craving after scanning (R = -0.222; P < 0.001). Finally, the individual habituation slope in the left sensory thalamus was correlated with DASS subscales (Anxiety (R = -0.179; P = 0.029) and Stress (R = -0.151; P = 0.015)) and overall DASS (R = -0.142; P = 0.029).

**Table C.** Correlation between the self-reported psychological and behavioral parameters with habituation slopes for the regions with dynamic activity using Pearson’s correlation coefficient.

| Psychological/behavioral parameters | Habituation slope in left vCa | |  | Habituation slope in right vCa | |  | Habituation slope in right NAc | |  | Habituation slope in right vmPu | |  | Habituation slop in left Stha | |
| --- | --- | --- | --- | --- | --- | --- | --- | --- | --- | --- | --- | --- | --- | --- |
|  | *R* | *P-value* |  | *R* | *P-value* |  | *R* | *P-value* |  | *R* | *P-value* |  | *R* | *P-value* |
| DASS | -0.066 | 0.498 |  | -0.116 | 0.247 |  | -0.197 | 0.075 |  | -0.036 | 0.565 |  | -0.142 | **0.029** |
| DASS (Depression) | -0.153 | 0.430 |  | -0.046 | 0.350 |  | -0.228 | 0.094 |  | -0.080 | 0.639 |  | -0.070 | 0.066 |
| DASS (Anxiety) | 0.020 | 0.831 |  | -0.072 | 0.456 |  | -0.096 | 0.206 |  | 0.053 | 0.879 |  | -0.179 | **0.029** |
| DASS (Stress) | -0.021 | 0.386 |  | -0.189 | 0.100 |  | -0.187 | **0.029** |  | -0.047 | 0.332 |  | -0.151 | **0.015** |
| CES | -0.002 | **0.007** |  | -0.170 | **< 0.001** |  | -0.066 | **0.002** |  | -0.213 | **< 0.001** |  | -0.002 | 0.131 |
| FCQ-T | -0.225 | **< 0.001** |  | -0.197 | **< 0.001** |  | -0.180 | **0.001** |  | -0.251 | **< 0.001** |  | 0.000 | 0.231 |
| FCQ-T (Lack of control) | -0.135 | **0.001** |  | -0.158 | **0.001** |  | -0.097 | **0.002** |  | -0.241 | **< 0.001** |  | 0.027 | 0.297 |
| FCQ-T (Thoughts) | -0.084 | **0.001** |  | -0.175 | **< 0.001** |  | -0.138 | **0.001** |  | -0.169 | **< 0.001** |  | 0.006 | 0.202 |
| FCQ-T (Hedonic hunger) | -0.328 | **< 0.001** |  | -0.216 | **< 0.001** |  | -0.234 | **< 0.001** |  | -0.318 | **< 0.001** |  | -0.029 | 0.193 |
| FCQ-T (Emotions) | -0.191 | **0.013** |  | -0.108 | **0.032** |  | -0.138 | **0.040** |  | -0.042 | **0.027** |  | 0.114 | 0.784 |
| FCQ-T (Guilt) | -0.150 | **0.007** |  | -0.109 | **0.007** |  | -0.136 | **0.007** |  | -0.112 | **0.005** |  | -0.167 | 0.067 |
| FCQ-S | -0.169 | **0.001** |  | -0.117 | **0.001** |  | -0.065 | **0.010** |  | -0.300 | **< 0.001** |  | 0.009 | 0.418 |
| FCQ-S (Desire) | -0.057 | **0.030** |  | -0.025 | **0.024** |  | 0.029 | 0.115 |  | -0.202 | **< 0.001** |  | -0.002 | 0.612 |
| FCQ-S (Positive reinforcement) | -0.218 | **< 0.001** |  | -0.245 | **< 0.001** |  | -0.189 | **0.001** |  | -0.365 | **< 0.001** |  | -0.034 | 0.227 |
| FCQ-S (Negative reinforcement) | -0.162 | **0.004** |  | -0.021 | **0.021** |  | 0.052 | 0.090 |  | -0.226 | **< 0.001** |  | 0.084 | 0.799 |
| FCQ-S (Control) | -0.106 | **0.001** |  | -0.235 | **< 0.001** |  | -0.202 | **0.001** |  | -0.360 | **< 0.001** |  | -0.162 | 0.119 |
| FCQ-S (Physiological hunger) | -0.137 | **0.013** |  | 0.067 | 0.075 |  | 0.062 | 0.186 |  | -0.038 | **0.008** |  | 0.163 | 0.780 |
| Pre Craving (VAS) | 0.024 | 0.388 |  | -0.110 | 0.260 |  | 0.102 | 0.830 |  | -0.107 | 0.087 |  | 0.223 | 0.172 |
| Post Craving (VAS) | -0.122 | **0.028** |  | -0.219 | **0.014** |  | -0.132 | 0.066 |  | -0.222 | **0.001** |  | 0.135 | 0.694 |
| Post-Pre Craving (VAS) | -0.182 | **0.016** |  | -0.166 | **0.012** |  | -0.275 | **0.007** |  | -0.173 | **0.005** |  | -0.059 | 0.399 |
| **Abbreviations:** vCa, ventral caudate; NAc, nucleus accumbens; vmPu, ventromedial putamen; Stha, sensory thalamus; DASS, depression anxiety stress scales; CES, compulsive eating scale; FCQ-S, food craving questionnaire-state; FCQ-T, food craving questionnaire-trait; VAS, visual analogue scale. | | | | | | | | | | | | | | |

**Supplementary Tables**

| **Supplementary Table 1\|** Significant clusters for the whole brain response to food cue reactivity in food > neutral contrast. | | | | | | | |  |
| --- | --- | --- | --- | --- | --- | --- | --- | --- |
|  |  | **Peak activation** | | |  | |  | |
| **Label** | **Side** | **x** | **y** | **z** | **Number of voxels** | **t-value** | |  |
| Posterior orbitofrontal cortex | L | 18 | -6 | -24 | 852 | 6.65. | |  |
| Occipital polar cortex | R | -12 | 105 | 6 | 688 | 5.88 | |  |
| Medioventral fusiform gyrus | L | 39 | 75 | -18 | 359 | 5.68 | |  |
| Inferior semilunar lobule | R | -6 | 75 | -48 | 189 | 6.05 | |  |
| Medioventral fusiform gyrus | R | -30 | 75 | -15 | 174 | 6.12 | |  |
| Medioventral cingulate gyrus | L | 0 | 33 | 33 | 103 | 5.12 | |  |
| Lateral superior frontal cortex | L | 3 | -60 | 51 | 98 | 5.03 | |  |
| Cerebellar tonsil | L | 15 | 33 | -51 | 92 | 5.26 | |  |
| Dorsal cingulate gyrus | L | 3 | 48 | 24 | 85 | 4.47 | |  |
| Inferior parietal lobule | L | 66 | 30 | 27 | 77 | -6.26 | |  |
| Lateral superior frontal gyrus | R | -21 | -36 | 66 | 74 | 5.49 | |  |
| Ventral middle frontal gyrus | R | -54 | -48 | 21 | 53 | 5.79 | |  |
| Rostral superior parietal lobule | R | -24 | 69 | 63 | 53 | 4.25 | |  |
| *Note.* Whole-brain activations are corrected for multiple comparisons using a cluster-based approach with a voxel-wise bi-sided *p*-value threshold of *p* < 0.0039 and a minimum cluster size of k = 50, which corresponds to a cluster-level alpha of *p* < 0.05 using NN2 clustering. **Abbreviations:** L, left; R, right. | | | | | | | |  |

| **Supplementary Table 2\|** Significant clusters for the main effects of *Time*, *Condition*, and *Time*$\times$*Condition* interaction on food cue reactivity related BOLD activation. | | | | | | | |  |
| --- | --- | --- | --- | --- | --- | --- | --- | --- |
|  |  | **Peak activation** | | |  | |  | |
| **Label** | **Side** | **x** | **y** | **z** | **Number of voxels** | **χ^2^(2)** | |  |
| *Main effect of Time* |  |  |  |  |  |  | |  |
| Occipital polar cortex | R | -15 | 99 | 6 | 1835 | 60.42 | |  |
| Dorsal agranular insular gyrus | L | 33 | -15 | -3 | 107 | 22.63 | |  |
| Superior temporal gyrus | L | 63 | 6 | 9 | 79 | 32.69 | |  |
| Middle frontal gyrus | R | -24 | -51 | 15 | 61 | 23.64 | |  |
| Precuneus | L | 0 | 78 | 51 | 55 | 31.30 | |  |
| Ventromedial putamen | R | -24 | -18 | -3 | 43 | 24.75 | |  |
| Posterior superior temporal sulcus | L | 57 | 30 | 6 | 40 | 33.28 | |  |
|  |  |  |  |  |  |  | |  |
| *Main effect of Condition* |  |  |  |  |  |  | |  |
| Precuneus | R | -6 | 75 | 27 | 169 | 14.43 | |  |
| Medioventral fusiform gyrus | L | 30 | 60 | -18 | 118 | 15.57 | |  |
| Superior parietal lobule | R | -27 | 63 | 48 | 104 | 11.46 | |  |
| NAc | R | -21 | -3 | -9 | 58 | 12.82 | |  |
| Cingulate gyrus | L | 0 | 36 | 33 | 58 | 10.85 | |  |
| Parahippocampal gyrus | R | -27 | 51 | -9 | 48 | 15.03 | |  |
|  |  |  |  |  |  |  | |  |
| *Time*$\times$*Condition interaction* |  |  |  |  |  |  | |  |
| Precuneus | R | 57 | -24 | 66 | 42 | 20.01 | |  |
| Occipital polar cortex | L | 54 | 18 | 96 | -6 | 30.90 | |  |
| Medioventral fusiform gyrus | L | 45 | 27 | 57 | -18 | 21.67 | |  |
| *Note.* Main effect of *Time* on whole-brain activation is corrected for multiple comparisons using a cluster-based approach with a voxel-wise bi-sided *p*-value threshold of *p* < 0.0033 and a minimum cluster size of k = 40, which corresponds to a cluster-level alpha of *p* < 0.05 using NN2 clustering. The effects of *Condition* and *Time × Condition* interaction on the whole-brain functional activation were analyzed with voxel-wise p-value thresholds of p < 0.05 and p < 0.01, respectively. χ^2^(2), peak chi-square value with two degrees of freedom. **Abbreviations:** NAc, Nucleus accumbens; L, left; R, right. | | | | | | | |  |

**Supplementary Figures**

**
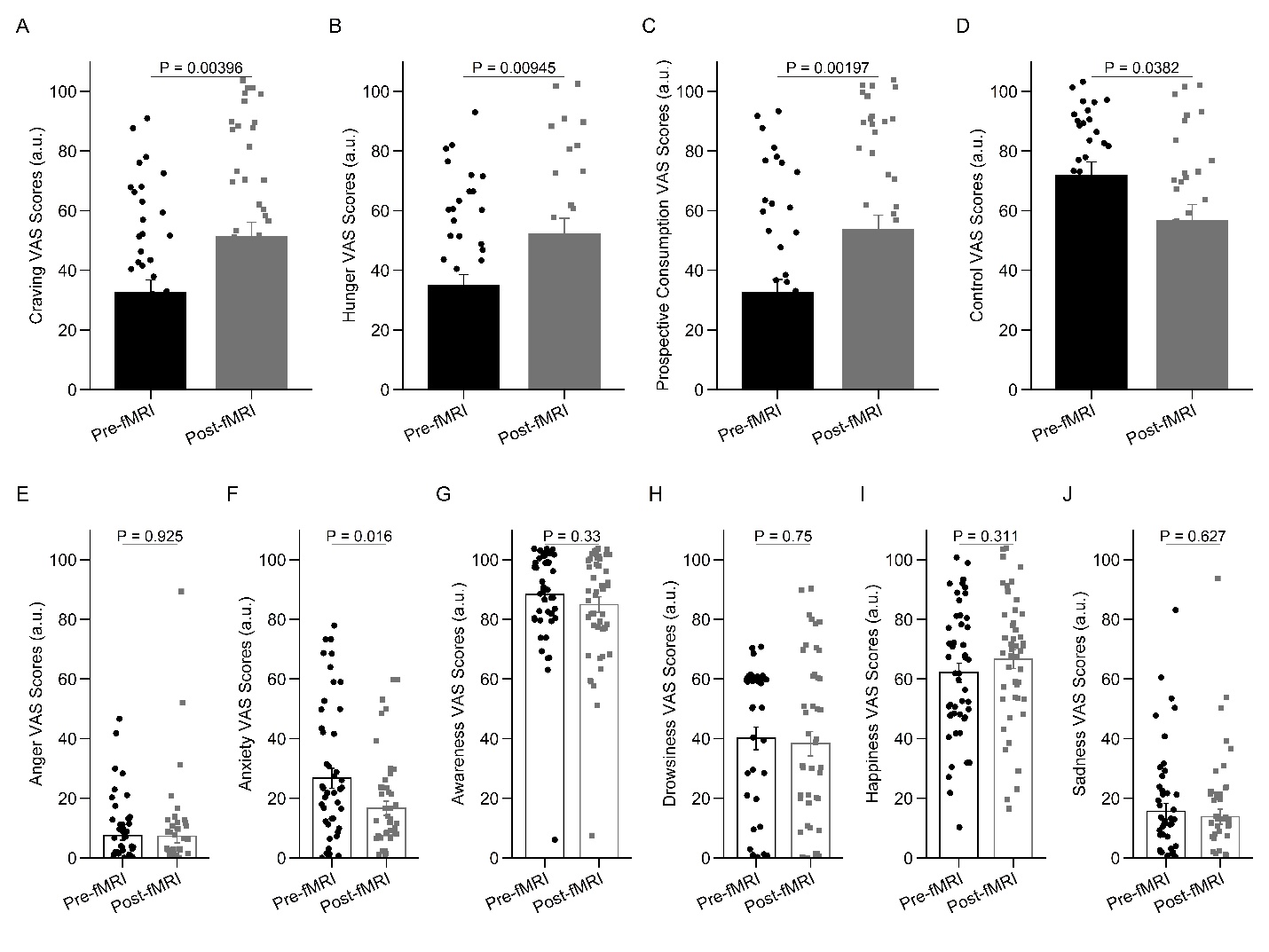
Supplementary Figure 1| Behavioral effects of food cue reactivity**. **(A–D)** Representative bar charts showing main effect of cue-reactivity on **(A)** craving; **(B)** hunger; **(C)** prospective consumption; and **(D)** food control before and after food cue-reactivity paradigm. **(E–J)** Changes in self-reported score of affective states (anger **(E)**, anxiety **(F)**, awareness **(G)**, drowsiness **(H)**, happiness **(I)**, and sadness **(J)**) before and after food cue-reactivity paradigm. Data in bar charts are represented as mean ± SEM.


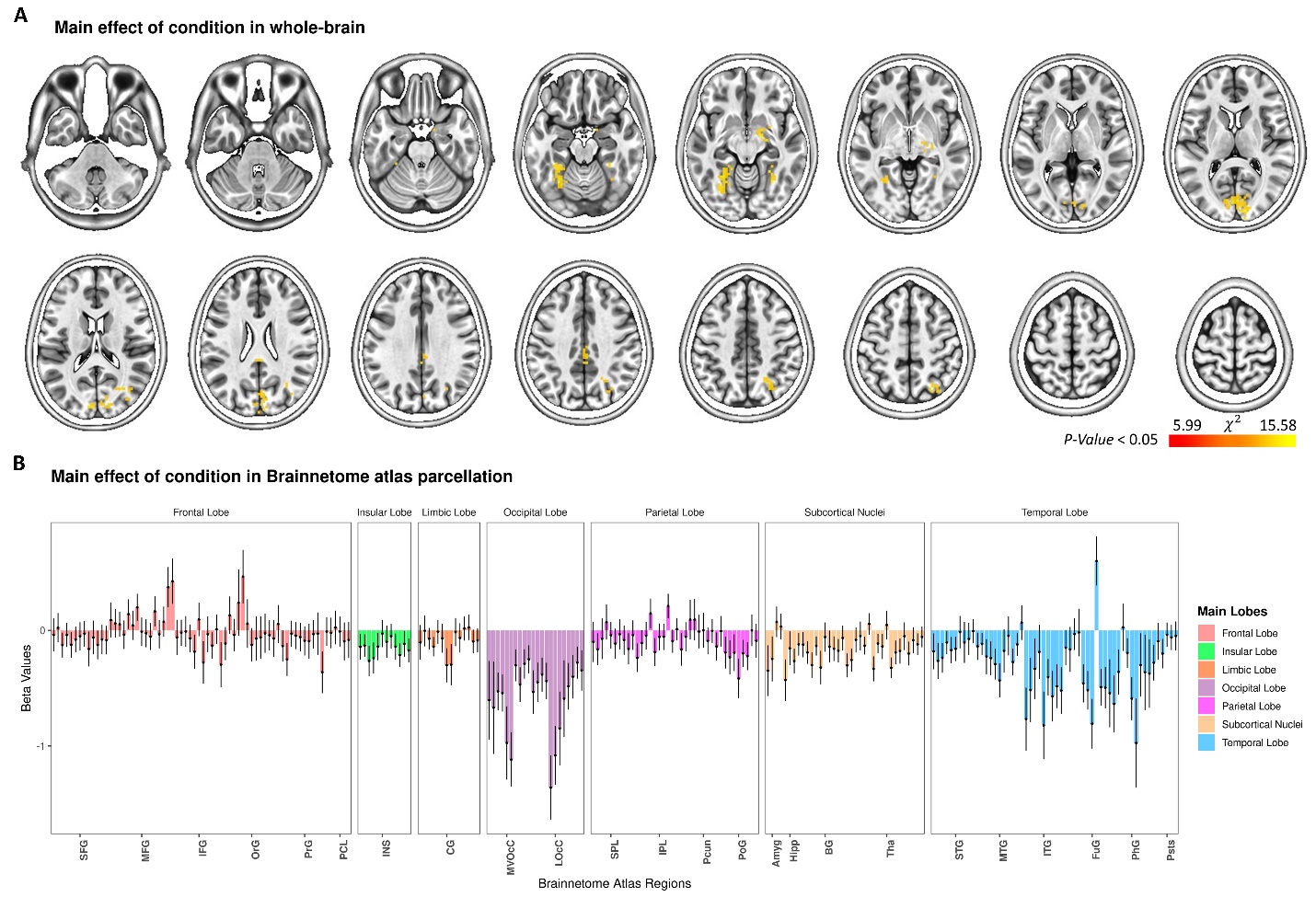
**Supplementary Figure 2| Change in functional brain activation during the fMRI food cue-reactivity task.** Main effect of *Condition* on the whole brain functional activation **(A)** and on Brainnetome atlas regions **(B)**. Data in bar charts are represented as mean ± s.e.m. **Abbreviations:** SFG, superior frontal gyrus; MFG, middle frontal gyrus; IFG: inferior frontal gyrus; OrG: orbital gyrus; PrG, precentral gyrus; PCL, paracentral lobule; STG, superior temporal gyrus; MTG, middle temporal gyrus; ITG, inferior temporal gyrus; FuG, fusiform gyrus; PhG, parahippocampal gyrus; pSTS, posterior superior temporal sulcus; SPL, superior parietal lobule; IPL, inferior parietal lobule; Pcun, precuneus; PoG, postcentral gyrus; INS, insular gyrus; CG, cingulate gyrus; MVOcC, medioventral occipital cortex; LOcC, lateral occipital cortex; Amyg, amygdala; Hipp, hippocampus; BG, basal ganglia; Tha, thalamus.


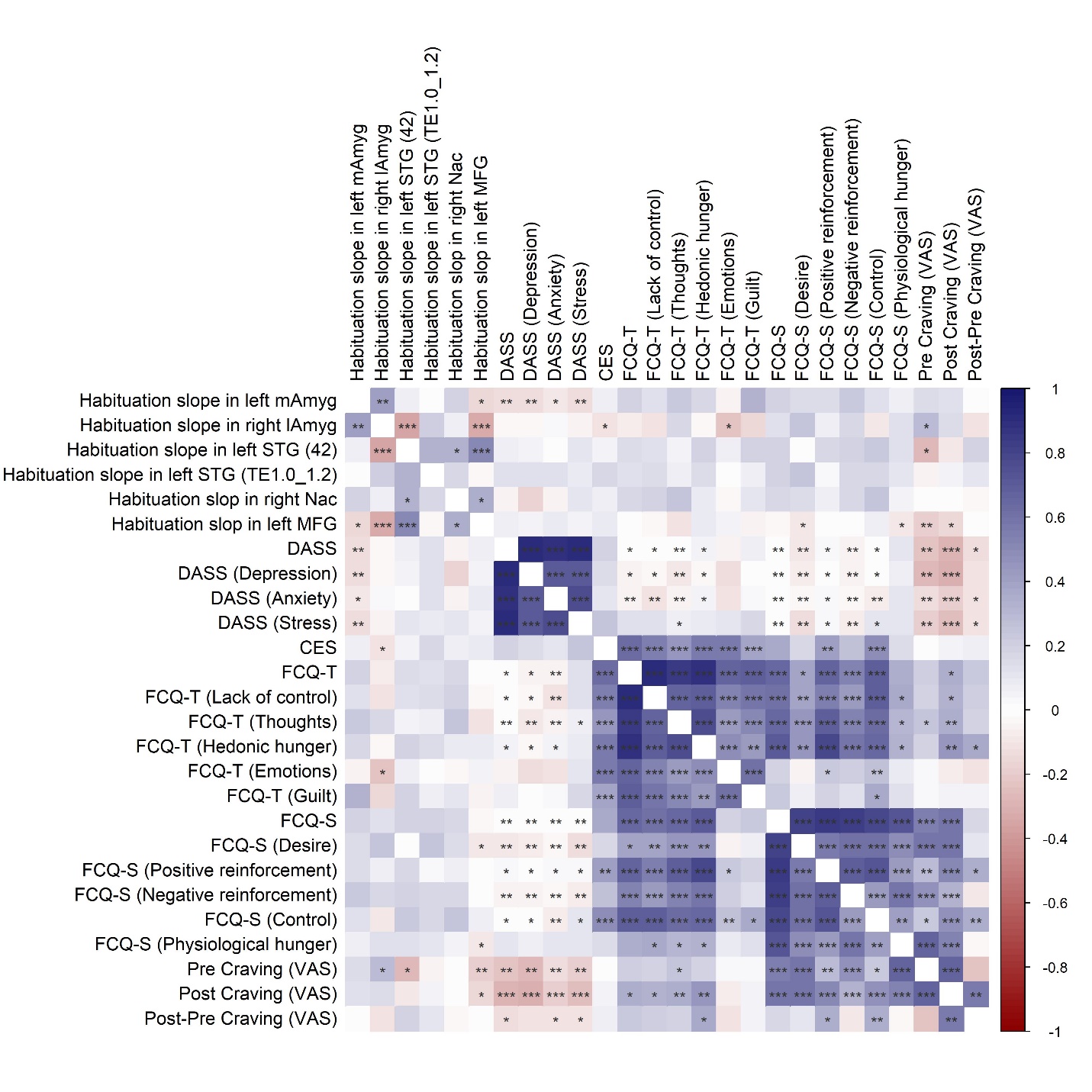
**Supplementary Figure 3| Brain-behavior relationships.** Correlation matrix between self-report psychological measures (DASS, DASS subscales, CES), behavioral measures (FCQ-Trait, FCQ-Trait subscales, FCQ-State, FCQ-State subscales, craving self-reports), and individual habituation slopes within the six regions of interest (left mAmyg, right lAmyg, left STG (42), left STG (TE1.0_1.2), right NAc, left MFG). Significant levels (**p* < 0.05, ***p* < 0.01, ****p* < 0.001). **Abbreviations:** mAmyg, medial amygdala; lAmyg, lateral amygdala; STG, superior temporal gyrus; NAc, nucleus accumbens; MFG, medial frontal gyrus; DASS, depression anxiety stress scales; CES, compulsive eating scale; FCQ-S, food craving questionnaire-state; FCQ-T, food craving questionnaire-trait; VAS, visual analogue scale.

**References:**

Avery, S. N., and Blackford, J. U. (2016). Slow to warm up: the role of habituation in social fear. *Soc. Cogn. Affect. Neurosci.* 11, 1832–1840. doi: 10.1093/scan/nsw095.

Plichta, M. M., Grimm, O., Morgen, K., Mier, D., Sauer, C., Haddad, L., et al. (2014). Amygdala habituation: A reliable fMRI phenotype. *NeuroImage* 103, 383–390. doi: 10.1016/j.neuroimage.2014.09.059.
